# Editorial: C1 inhibitor deficiency and angioedema

**DOI:** 10.3389/falgy.2022.1065400

**Published:** 2022-11-09

**Authors:** Henriette Farkas, Anastasios E. Germenis, Hilary Longhurst

**Affiliations:** ^1^ Department of Internal Medicine and Haematology, Hungarian Angioedema Center of Reference and Excellence, Semmelweis University, Budapest, Hungary; ^2^ Department of Immunology & Histocompatibility, School of Health Sciences, Faculty of Medicine, University of Thessaly, Larissa, Greece; ^3^ Department of Immunology, Auckland District Health Board, and Department of Medicine, University of Auckland, Auckland, New Zealand

**Keywords:** hereditary angioedema (HAE), C1 inhibitor deficiency, bradykinin, *SERPING1* gene, plasma-derived C1-inhibitor

**Editorial on the Research Topic** C1 inhibitor deficiency and angioedema By Farkas H, Germenis AE, Longhurst H. (2022) Front. Allergy. 3: 1065400. doi: 10.3389/falgy.2022.1065400

The turn of the century was followed by a rapid expansion of our knowledge about C1-inhibitor (C1-INH) and angioedema. The role of C1-INH as the most important inhibitor in the kinin-forming cascade was further elucidated along with many other aspects of the production and regulation of bradykinin; genomics allowed a faster and more integrated analysis of the *SERPING1* gene as well as the recognition of five genes –beyond the *F12* gene– associated with angioedema with normal C1-INH (HAE-nC1-INH); and an abundance of therapeutic modalities were developed, perhaps higher than any other rare disease.

In the 23 years since its inception, the international C1 inhibitor Deficiency and Angioedema Workshop has been the premier forum for the presentation and discussion of this remarkable progress. Nine representative works presented in the most recent 12th C1 inhibitor Deficiency and Angioedema Workshop, held virtually, are included in this Research Topic.

In a detailed review article Kaplan et al. present the complex role of kininogens, and the substrates from which bradykinin is derived in hereditary angioedema (HAE), focusing on the multifaceted mechanisms by which high molecular weight kininogen simultaneously augments all the steps required for bradykinin formation and intrinsic coagulation and fibrinolysis, beyond being a substrate from which bradykinin is generated. These interacting mechanisms are intimately involved in manifestations of hereditary angioedema caused by C1 inhibitor deficiency, and beyond.

The diagnosis of hereditary angioedema is still challenging, especially in regard with special patient groups and/or the use of emerging approaches, i.e., genetics. Andrási et al. summarize their experience in the diagnostics of C1-inhibitor deficiency in pediatric age. The study presents a strategy for monitoring children in an Angioedema Center, emphasizes the importance of screening and performing complement and DNA tests not just in peripheral blood, but also in cord blood to allow the early and proper diagnosis before the onset of clinical manifestations, even in cases with misleading complement result. Early diagnosis provides an opportunity to supply the patient with the appropriate treatment, as HAE attacks can occur at any age and their onset cannot be predicted in advance.

The wide and easy application of genomic techniques made intriguing the role of genotyping in the diagnosis of HAE due to C1-deficiency (HAE-C1-INH). Szabó et al. present their strategy on genetic work-up exploring the *SERPING1* gene and provide an overview of the mutations identified in a large cohort of Hungarian HAE-C1-INH patients. The combination of conventional and novel methods allowed them to unravel the genetic cause behind C1-inhibitor deficiency in each affected pedigree involving the identification of five, previously unreported variations. Detection and correct interpretation of disease causing *SERPING1* variants is of great importance in HAE-C1-INH as by facilitating correct and early diagnosis the patient's proper treatment, prognosis and quality of life can be improved.

Recognizing C1-INH features and *SERPING1* genetics together is a prerequisite for the curation of variant pathogenicity. With this aim, Drouet et al. reviewed all 809 reported variants of the SERPING1 gene in relation to the biological and structural features of C1-INH. This is largest study of the constellation of *SERPING1* variants found in nearly 1,500 HAE families, emphasizing that etiopathogenesis of HAE-C1-INH can be consistently predicted by C1-INH molecular analyses.

In regard with the diagnosis and management of HAE, pregnancy remains an interesting aspect of the disease. Considering the strong relevance of estrogens in HAE-nC1-INH, Gabriel et al. investigated the history of 45 pregnancies occurring in 26 HAE-nC1-INH patients. They found that the occurrence of abortion in HAE-nC1-INH was similar the expected rate for unaffected women. However, the first trimester of the pregnancy was more symptomatic for HAE-nC1-INH women. The authors concluded that although pregnancy could not be inputted as more dangerous for women with HAE-nC1-INH than the disease *per se*, a multidisciplinary approach, involving the obstetrician and other health care professionals when needed, would be beneficial.

The great variability of the clinical expression of HAE remains a serious, yet unmet problem. Serious efforts are made towards detecting biochemical and/or genetic biomarkers for diagnostic, prognostic and preventive use. Using a liquid chromatography coupled with tandem mass spectrometry (LC-MS/MS) platform of picomolar sensitivity developed for the analysis of eleven bradykinin (BK)-related peptides, Marceau et al. measured the presence of BK-related peptides in the plasma of patients with HAE-C1INH and HAE-FXII during remission, in order to examine whether some of these peptides might be biomarkers of these forms of the disease. According to their results, the concentrations of BK_1–5_, BK_2–9_ and the sum of BK and its fragments could be used as biomarkers of HAE-C1-INH but not of HAE-FXII in remission.

On the other hand, accumulating evidence indicates that clinical variability of HAE-C1-INH is substantially attributable to modifier genes. To further examine this hypothesis, Parsopoulou et al. investigated the presence or absence of 18 functional variants of genes encoding proteins involved in the metabolism and function of bradykinin, in relation to three distinct phenotypic traits of patients with HAE-C1-INH, i.e., the age at disease onset, the need for long-term prophylaxis (LTP), and the severity of the disease. Their findings confirmed that variants other than the *SERPING1* causal variants, like *F13B-rs6003, PLAU-rs2227564, SERPINA1-rs28929474, SERPINA1-rs17580, KLK1-rs5515, SERPINE1-rs6092*, and *F2-rs1799963*, act as independent modifiers of HAE-C1-INH severity and could be tested as possible prognostic biomarkers.

Finally, as the treatment of HAE is considered, in their review article, Valerieva and Longhurst compare C1-INH replacement with newer therapies targeting the contact pathway. Both approaches have been shown to be effective for acute treatment and prophylaxis; with approved and investigational therapies showing therapeutic efficacy of inhibition of a number of targets in the contact pathway. These include (pre)kallikrein, the bradykinin B2 receptor and activated factor XII. A variety of therapeutic mechanisms, including small molecules, monoclonal antibodies, RNA silencing and genetic therapies are available or in development.

Accordingly, in their case series study, Zanichelli et al. report their real-life experience from HAE-C1-INH patients poorly controlled with their previous long-term prophylaxis or with difficult venous access, who received subcutaneous plasma-derived C1-inhibitor (pdC1-INH) at lower than the recommended doses. Their results indicate that, in patients with difficult venous access, in countries where pdC1-INH is not approved for subcutaneous administration, about half the recommended dose may be beneficial, although suboptimal results may be obtained, compared with licensed doses.

We hope that this Research Topic will provide a brief indication of the current state of the art and suggestions for further discussion during the forthcoming 13th C1 inhibitor Deficiency and Angioedema Workshop.

